# Effects of Cryoconcentrated Blueberry Juice as Functional Ingredient for Preparation of Commercial Confectionary Hydrogels

**DOI:** 10.3390/gels8040217

**Published:** 2022-04-01

**Authors:** Nidia Casas-Forero, Igor Trujillo-Mayol, Rommy N. Zúñiga, Guillermo Petzold, Patricio Orellana-Palma

**Affiliations:** 1 Programa de Doctorado en Ingeniería de Alimentos, Facultad de Ciencias de la Salud y de los Alimentos, Campus Fernando May, Universidad del Bío-Bío, Av. Andrés Bello 720, Chillán 3780000, Chile; igor.trujillo@gmail.com; 2 Laboratorio de Crioconcentración, Departamento de Ingeniería en Alimentos, Facultad de Ciencias de la Salud y de los Alimentos, Campus Fernando May, Universidad del Bío-Bío, Av. Andrés Bello 720, Chillán 3780000, Chile; gpetzold@ubiobio.cl; 3 Departamento de Biotecnología, Facultad de Ciencias Naturales, Matemática y del Medio Ambiente, Campus Macul, Universidad Tecnológica Metropolitana, Las Palmeras 3360, Ñuñoa, Santiago 7800003, Chile; rommy.zuniga@utem.cl; 4 Programa Institucional de Fomento a la Investigación, Desarrollo e Innovación, Universidad Tecnológica Metropolitana, Ignacio Valdivieso 2409, San Joaquín, Santiago 8940577, Chile; 5 Grupo de Crioconcentración de Alimentos y Procesos Relacionados, Universidad del Bío-Bío, Av. Andrés Bello 720, Chillán 3780000, Chile; 6 Departamento de Ingeniería en Alimentos, Facultad de Ingeniería, Campus Andrés Bello, Universidad de La Serena, Av. Raúl Bitrán 1305, La Serena 1720010, Chile

**Keywords:** cryoconcentrated blueberry juice, hydrogels, stability, storage, bioactive compounds content, antioxidant activity

## Abstract

Hydrogels can absorb and/or retain components in the interstitial spaces due to the 3D cross-linked polymer network, and thus, these matrices can be used in different engineering applications. This study focuses on the physicochemical and textural properties, as well as bioactive compounds and their antioxidant activity stability of commercial hydrogels fortified with cryoconcentrated blueberry juice (CBJ) stored for 35 days. CBJ was added to commercial hydrogels (gelatin gel (GG), aerated gelatin gel (AGG), gummy (GM), and aerated gummy (AGM)). The samples showed a total polyphenol, anthocyanin, and flavonoid content ranging from 230 to 250 mg GAE/100 g, 3.5 to 3.9 mg C3G/100 g, and 120 to 136 mg CEQ/100 g, respectively, and GG and GM showed the lowest bioactive component degradation rate, while AGM presented the highest degradation. GG and GM samples could be stored for up to 21 days without significant changes, while the results indicated ≈15 days for the AGG and AGM samples. Thereby, CBJ offers enormous possibilities to be used as a functional ingredient due to the high nutritional values, and it allows enriching different hydrogel samples, and in turn, the structures of hydrogels protected components during in vitro digestion, enhancing the bioaccessibility after the digestion process.

## 1. Introduction

Confectionery hydrogel products have a high popularity and demand among consumers (mainly children and the elderly) due to their visual attractiveness, texture, and mouthfeel [1]. Specifically, the confectionery hydrogel products are manufactured with gelling agents, sucrose, glucose syrup, acids, flavorings, and colorants [2]. Thus, depending on the type of processing and mixture of ingredients, it is possible to obtain hydrogel products with different textures, including gelatin gels with soft texture [3], gummy characterized by a firm, soft and chewy texture structure [4], and marshmallows or aerated gummy (conferred by air bubbles) with a soft and elastic texture [5].

On the other hand, the growing awareness and knowledge (individual and community) among consumers have encouraged food industries to replace ingredients harmful to human health used in many food products. Whereby confectionery hydrogel products have been questioned due to their high sugar content and low nutritional value. This combination has been associated with obesity, diabetes, cardiovascular diseases, and hypertension [6]. Thus, consumers demand healthier confectionery hydrogels foods with lower sugar content and higher natural antioxidants than current commercial products [7]. Therefore, researchers are endeavoring to improve the nutritional value and consumer appreciation of these products. The most common strategies are based on sugar substitution through low-caloric sweeteners, such as sorbitol, isomaltulose, and stevia [6,8,9]. Hence, various bioactive compounds have been incorporated into hydrogels products, such tea extract [7,10], hibiscus extract [11], betalain-rich extract [12], watermelon juice [13], pomegranate juice [14], and cryoconcentrated blueberry juice [15,16].

In particular, cryoconcentrated juices retain their natural sugars, bioactive compounds, and antioxidant activity due to low-temperature processing through cryoconcentration technology [17], display attractive colors, and have interesting health benefits [18,19,20]. However, it is essential to understand that these bioactive compounds could lose their beneficial bioactivity during storage due to their instability under various ambient factors [21,22]. Therefore, it is important to establish their stability through different storage conditions. In this sense, kinetic models have been used to predict the influence of storage on the stability of bioactive compounds [21]. Tutunchi et al. [22] and Rodríguez-Sánchez et al. [23] reported that betanin and betaxanthins’ degradation in gummy candy considers first-order kinetics. However, there are few studies on the degradation kinetics of bioactive compounds in commercial hydrogels based on confectionery products during storage conditions, which is important for the food industry due to their interest in the development of confectionery products with health-promoting effects.

Therefore, the aim of this study was to evaluate physicochemical and textural properties, stability of the bioactive compound, and antioxidant activity in commercial hydrogel products based on confectionery foods fortified with cryoconcentrated blueberry juice during storage conditions (4 °C and 25 °C) over 35 days.

## 2. Results and Discussion

### 2.1. Characterization of Hydrogels Samples

#### 2.1.1. Physicochemical Properties

Table 1 summarizes the physicochemical properties of the samples. 

Initially, GG and AGG reached a TSS content of around 17 °Brix and 21 °Brix, respectively. Rubio-Arraez et al. [3] reported a similar TSS value (20 °Brix) in gels with citrus juice and non-cariogenic sweeteners. Thus, this result means that CBJ provides the TSS necessary to obtain gelatin gel without sweeteners. While GM and AGM exhibited lower TSS values (≈60 °Brix) than that indicated by studies in gummies and marshmallows [10,11,24,25], which reported values between 70 °Brix and 80 °Brix in the hydrogel samples. This difference may be due to the partial substitution of the syrup for CBJ, resulting in GM and AGM with a lower amount of added sugar. For pH, the values ranged from 5.0 to 5.2. These values are lower than those previously reported for similar products, ranging from 6.0 and 6.9 [5,24,25]. This decrease in pH value can be related to the CBJ added in the samples, which provides organic acids, such as malic, citric, and shikimic acids [26]. In the same way, the moisture content and a_w_ in the GG and AGG samples were in agreement with the values reported by Rubio-Arraez et al. [3]. For GM and AGM hydrogels, the results were significantly higher than those stated by Mardani et al. [25] and Periche et al. [5,24], who observed a moisture content value close to 16% to 24% and a_w_ of approximately 0.75 to 0.85. In contrast, Mandura et al. [10] and Šeremet et al. [7] reported high moisture content (34%) for gummy with a white tea infusion as an ingredient. Therefore, these differences could be attributed to TSS and phenolic compounds since the phenolic groups can hold more water due to interactions between the hydroxyl groups of polyphenols, proteins, sugars, and water [27].

For density, the aerated samples (AGG and AGM) showed a reduction of 35% compared to the non-aerated samples (GG and GM) due to the incorporation of air bubbles [15]. This incorporated air (ε) corresponds to 62% and 66% for AGG and AGM, respectively. These results are in line with the values reported by Casas-Forero et al. [15] and Mardani et al. [25] for aerated gelatin gel and marshmallows, respectively. Furthermore, air is the “main ingredient” of aerated confectionery, and it provokes a significant decrease in density [28].

#### 2.1.2. Rheological and Mechanical Properties

**Figure 1:**
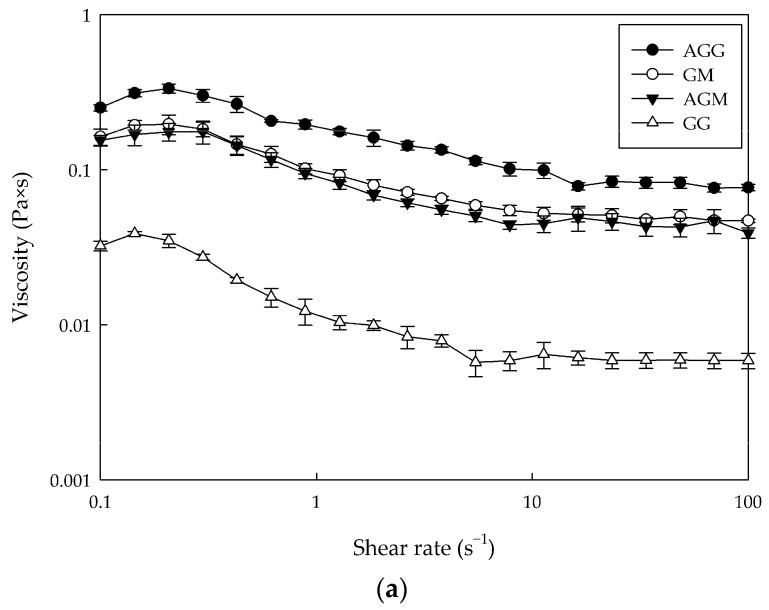

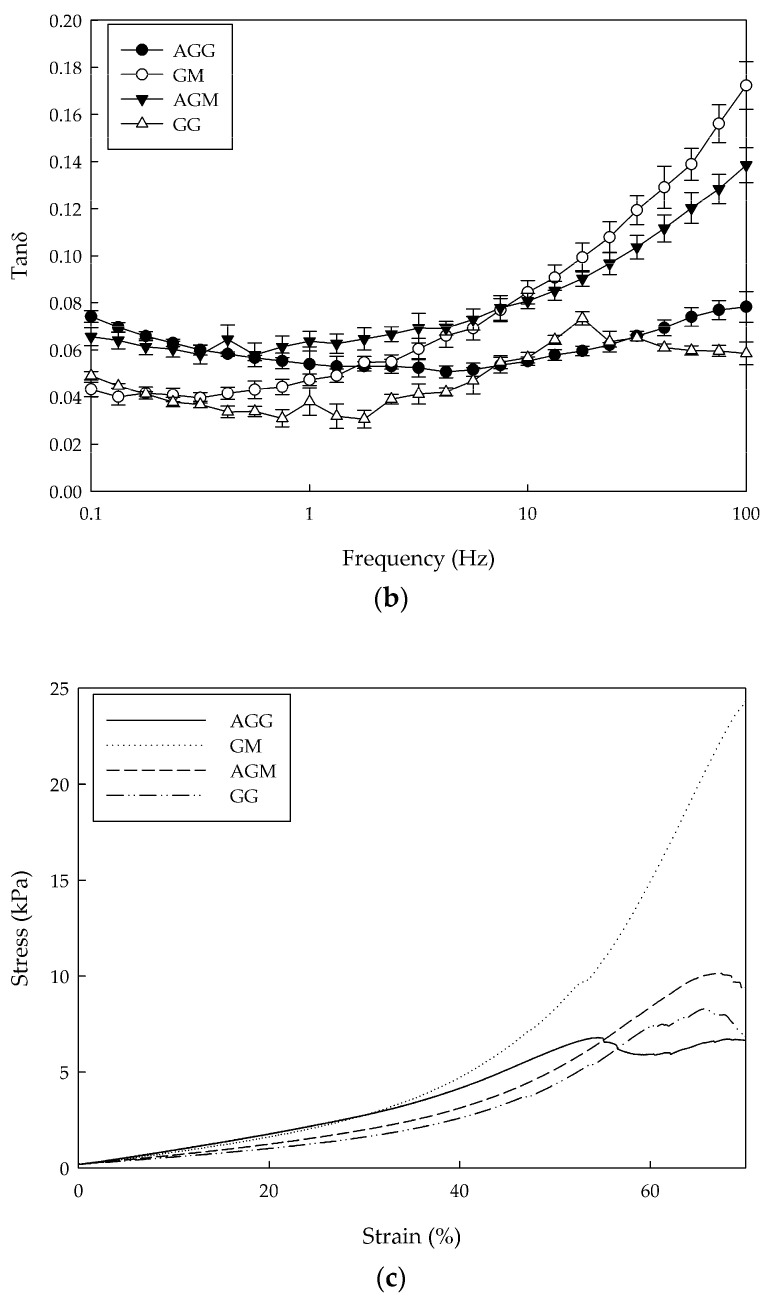
Rheological and mechanical properties of hydrogel samples: (**a**) The changes in viscosity during shear rate; (**b**) The changes in tan δ (G″/G′) during frequency scanning; (**c**) Stress and strain curves in uniaxial compression.

Figure 1 shows the rheological behavior of the hydrogel samples.

First, all samples exhibited similar behavior. Thus, at low shear rates (<10 s^−1^), the viscosity decreased with an increasing shear rate, indicating that the samples had a pseudoplastic fluid and shear thinning behavior. Then, at high shear rates (>10 s^−1^), the viscosity no longer changed with the increase in shear rate (Newtonian fluid) [6]. Similarly, Figure 1a (relationship between apparent viscosity and shear rate) shows that GG had the lowest viscosity (0.006 Pa·s). In contrast, AGG significantly increased its viscosity (0.083 Pa·s), showing values close to those found for GM and AGM (≈0.05 Pa·s). This increase in viscosity is mainly related to an increase in the amount of gelatin added in the AGG, GM, and AGM samples, which was significantly higher than GG hydrogel, with an increase of 2.6, 2.0, and 2.0 times, respectively. This behavior agrees with that reported by Casas-Forero et al. [15]. Furthermore, as described above, aeration in confectionery changes its physical properties, in particular, an increase in viscosity and a decrease in fluidity [28]. 

In the same way, the value of tan δ (G″/G′) is a parameter of the viscoelastic behavior of the materials [29]. As shown in Figure 1b, the tan δ of all samples was less than 0.5 in the entire frequency range, indicating that an elastic behavior predominated over the viscous, which is characteristic of these types of hydrogel products [6].

Additionally, stress-deformation curves of the samples during uniaxial compression are depicted in Figure 1c. All samples exhibited a sigmoid stress–strain behavior, characteristic of foam materials [30]. As can be seen, GG had fracture stress and strain of 8 kPa and 65%, respectively, exhibiting the characteristics of a soft material. The results are consistent with the literature, which reports 4 kPa to 8 kPa and 60% values for fracture stress and strain, respectively [31,32]. AGG was characterized by a weaker and less ductile texture with 6 kPa for fracture stress and 55% for fracture strain. This difference between GG and AGG is attributed to the air bubbles, which reduce the matrix content per unit of sectional [32]. This is in line, with Hartel et al. [28] who indicated that the texture properties of aerated confectionery are largely dependent on the air phase. For GM and AGM, fracture stress and strain values in GM (23 kPa and 69%, respectively) were higher than achieved by AGM (10 kPa and 67%, respectively). This means that GM had a firm and elastic texture, while AGM was a product with a soft elastic texture. Likewise, AGM showed fracture stress close to those obtained in a commercial marshmallow (9 kPa) [33], indicating that it is possible to develop hydrogel foods with CBJ as a source of natural sugar and bioactive compounds without affecting its texture, which would be a potential commercial advantage. 

#### 2.1.3. Microstructural Features

**Figure 2:**
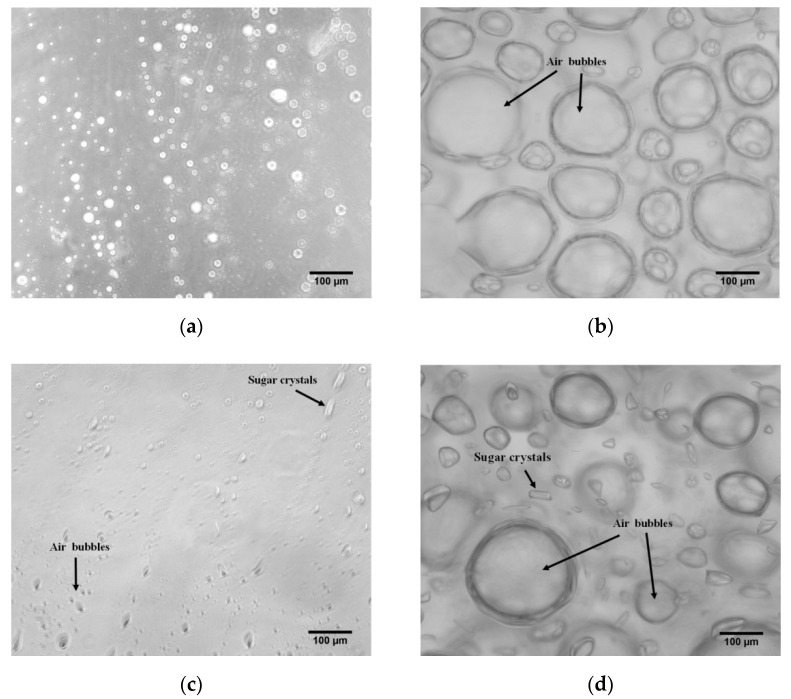
Optical micrograph images of gelatin-based confectionery at 10X: (**a**) GG; (**b**) AGG; (**c**) GM; (**d**) AGM.

Micrographs of the confectionery are shown in Figure 2. 

GG exhibited a homogeneous structure with a small strand formation (Figure 2a), which is characteristic of the microstructure of bovine gelatin [12]. GM preserved the uniform structure of GG with the formation of some air bubbles and any tiny sugar crystals (Figure 2c). A similar microstructure was reported by de Moura et al. [11] and Kumar et al. [34] in jelly candies with hibiscus extract-encapsulated and vegan gummy candy, respectively. In the case of the aerated samples, AGG and AGM showed small and large air bubbles. Large bubbles with a marked spherical shape were characterized in AGG hydrogels (Figure 2b), while AG showed small bubbles with non-spherical shapes and the formation of tiny sugar crystals (Figure 2d). The difference in bubble size between AGG and AGM can be attributed to the gelatin content, as evidenced by Casas-Forero et al. [15] in the study of aerated gelatin gels that increased small bubbles as the amount of gelatin decreased. 

### 2.2. Stability of Hydrogel Samples during Storage

#### 2.2.1. Stability of TBC Content and Antioxidant Activity

Total bioactive compounds (TBC) and antioxidant activity (AA) values in the initial sample and hydrogel samples are shown in Table 2. 

The fortification of food with juice rich in bioactive compounds can improve the nutritional and functional values of these products [35]. First, CBJ had a TBC content close to 770 mg GAE/100 g for TPC, 22 mg C3G/100 g for TAC, and 560 mg CEQ/100 g for TFC, and the AA values were approximately 4585 μmol TE/100 g for DPPH and 4440 μmol TE/100 g for FRAP.

On day 0, hydrogel samples displayed a TBC content ranging from 230 to 250 mg GAE/100 g for TPC, 3.5 to 3.9 mg C3G/100 g for TAC, and 120 to 136 mg CEQ/100 g for TFC. These slight variations in TBC content between the samples could be due to processing conditions since all samples were fortified with CBJ at 30% (*w*/*w*). Compared to other studies, the TPC obtained was higher than those reported in white tea-based candies (170–180 mg GAE/100 g) [7], pomegranate juice-based candies (72–159 mg GAE/100 g) [14], and jelly candies with rosemary extract (227 mg GAE/100 g) [8]. In contrast, Rivero et al. [9] reported polyphenol content of over 50% in candies with raspberry juice powder (490–550 mg GAE/100 g). These differences between studies can be related to the formulation, and the process conditions used.

In terms of AA, all the samples exhibited similar FRAP values (1050–1085 μmol TE/100 g), while DPPH values varied between samples. Thus, in DPPH, GG and AGG had values of 780 and 760 μmol TE/100 g, respectively, while GM and AGM increased the DPPH results, reaching values of 910 and 870 μmol TE/100 g, respectively. The values are comparable to those reported by Rivero et al. [9], Mandura et al. [10], and Hani et al. [36], who studied the AA of gummy with red pitaya fruit puree, white tea-based candies, and jellies containing honey and propolis, respectively.

Additionally, we observed a proportional trend between CBJ and hydrogel samples. A decrease varying from ≈70% to ≈85% for TBC and DPPH values were observed, while FRAP decreased in all the samples (44.7% for GG, 24.5% for AGG, 39.5% for GM, and 19.1% for AGM). These variations are in agreement with the results reported by Cedeño-Pinos et al. [8], who suggested that the reduction in the AA level in gelatin-based candies may be due to interactions between ingredients, the presence of amino acids, or the interaction of antioxidants with the gelling matrix.

**Figure 3:**
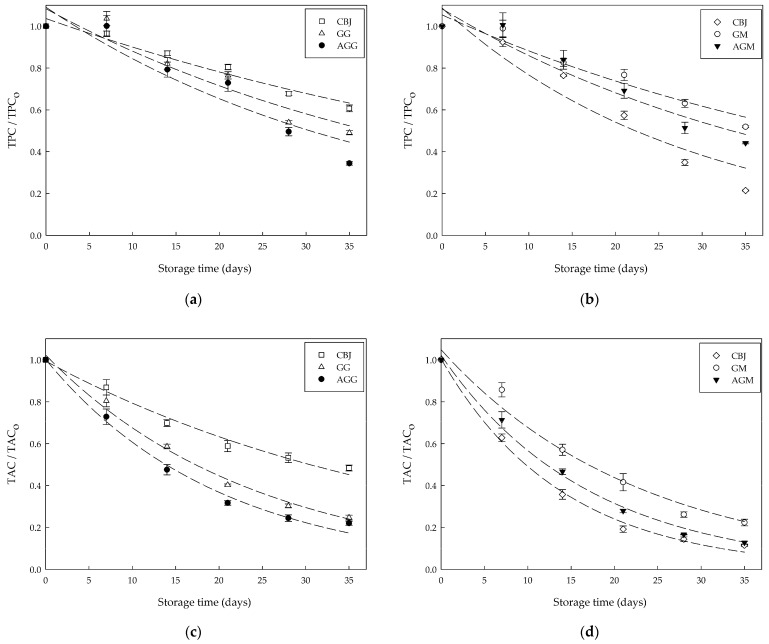

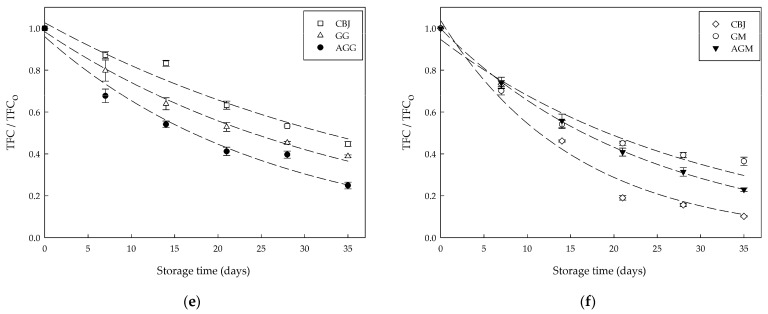
Degradation of total bioactive compounds of the gelatin-based confectionery and CBJ during storage: (**a**) TPC at 4 °C; (**b**) TPC at 25 °C; (**c**) TAC at 4 °C; (**d**) TPC at 25 °C; (**e**) TFC at 4 °C, (**f**) TPC at 25 °C.

Figure 3 shows the degradation of total bioactive compounds of the hydrogel samples and CBJ during storage. 

The CBJ showed a significant reduction in TBC by increasing the temperature from 4 to 25 °C during storage. This decrease in TBC was between 40% and 50% for CBJ stored at 4 °C and about 80% for CBJ stored at 25 °C. This result was higher than the previously reported value by Orellana-Palma et al. [19] for cryoconcentrated Calafate juice, who reported a loss between 4% and 25% in the TBC after 35 days of storage at 4 °C. Regarding anthocyanin stability studies in blueberry juice, Barba et al. [37] indicated a decrease of about 9% after 7 days of storage at 4 °C in juice treated with PEF. Furthermore, Cortellino and Rizzolo [38] and Zhang et al. [39] observed in thermally treated juice a loss of 60% after 150 days at 20 °C and 80% after ten days at 4 °C, respectively. These differences in the TBC degradation rate during storage can be related to pretreatments before juice storage that could inactivate enzymes, such as polyphenol oxidase and peroxidase, which are considered responsible for the decay of phenols in berry-derived foods [38].

Figure 3a,b show that TPC gradually decreased as the storage time increased. After 35 days, the polyphenols loss was 50% and 65% for GG and AGG, respectively, whereas GM and AGM showed a lower loss with 48% and 55% values. This difference between samples could be related to the moisture content and water activity, since a more significant volume of water favors reactant mobility, contributing to phenolic compounds degradation [40]. Maier et al. [41] also observed a decrease in the phenolic content from 243.6 mg/kg to 82.6 mg/kg in gelatin gels enriched with grape pomace extract during storage. Furthermore, Tutunchi et al. [22] indicated a loss of TPC of 25% to 35% for gummy candy with red beet extract powder at 28 days. Meanwhile, Šeremet et al. [7] reported fluctuations in the TPC during the storage of white tea-based candies.

On the other hand, TAC decreased markedly over 35 days, with a total decrease of 75%, 78%, 77%, and 87% for GG, AGG, GM, and AGM, respectively (Figure 3c,d). These results were consistent with the findings of de Moura et al. [11] and Maier et al. [41]. They reported a reduction of close to 70% in anthocyanin content in jelly candy with hibiscus extract and gelatin gels enriched with grape pomace extract, respectively. Tavares et al. [42] mentioned that anthocyanins are unstable and highly susceptible to degradation under conditions such as pH, temperature, light, oxygen, enzymes, ascorbic acid, and copigments. Likewise, Chen et al. [21] and Teribia et al. [43] suggested that anthocyanin degradation could result from condensation and polymerization reactions with other phenolic compounds present in the sample. Hence, this anthocyanin decrease can lead to undesirable color changes and a reduction in antioxidant activity.

Like the results of the TPC and TAC, the storage also had an unfavorable influence on the TFC (Figure 3e,f). At the end of 35 days of storage, the flavonoids content in GG and AGG stored at 4 °C decreased by approximately 60% and 75%, respectively, and for the GM and AGM samples stored at 25 °C, this reduction was up to 62% and 77%, respectively. In general terms, the stability of TBC in gelatin-based foods was slightly higher than CBJ mainly when stored at 25 °C, possibly due to the interaction of polyphenols with gelatin that led to lower availability of polyphenols for hydrolysis and oxidation reactions [22,23]. Similarly, Kia [44] indicated that the gelatin triple helix could retain antioxidant compounds due to crosslinking and the formation of three-dimensional networks. On the other hand, the TBC stability in samples containing air bubbles inside the food matrix (AGG and AGM) was significantly (*p* ≤ 0.05) lower than those samples non-aerated (GG and GM). This can be related to oxygen reacting with antioxidant compounds through hydrogen atom donation of the hydroxyl group to a free radical, leading to a decrease in the bioactive compounds [45].

**Figure 4:**
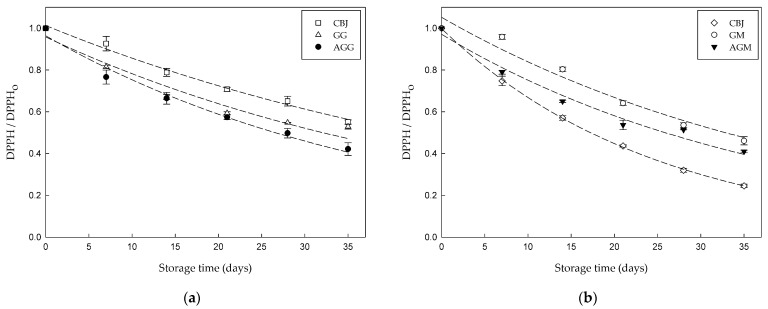

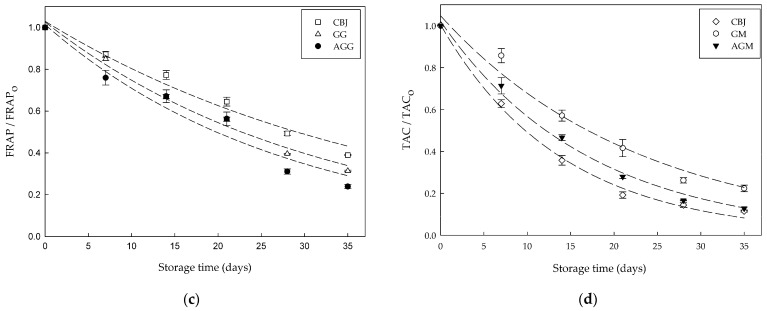
Antioxidant activity stability of hydrogel samples and CBJ during storage: (**a**) DPPH at 4 °C; (**b**) DPPH at 25 °C; (**c**) FRAP at 4 °C; (**d**) FRAP at 25 °C.

Figure 4 shows a significant reduction (*p* ≤ 0.05) in the values of DPPH and FRAP during the storage period at 4 and 25 °C. 

DPPH values of hydrogel samples decreased by approximately 54% by day 35. Meanwhile, a higher loss was observed in the FRAP assay, which decreased between 65% and 75% by day 35. This reduction might be due to the degradation of bioactive compounds, mainly anthocyanins [21]. The decrease in AA during storage has been reported in other studies, including a decrease in AA of 64% in jelly prepared with citrus juice [3], 51% in white tea-based candies [10], 21% to 37% in gummy candy with red beet extract powder [22], 15% in vegan gummy candies with betalains nanoliposomes [34], and 55% to 70% in gummy candy with red beet extract [46].

On the other hand, k and t_1/2_ are summarized in Table 3. 

The TBC and AA degradation was appropriately fitted to a first-order kinetic model (R^2^ > 0.9), which is similar to the previous studies on the degradation of bioactive compounds in blueberries [41,47]. As expected, in CBJ samples, the storage temperature significantly affected the k values of TBC and AA. The results indicate that CBJ stored at 25 °C degrades three times faster than CBJ stored at 4 °C. Anthocyanins showed the most remarkable changes, decreasing t_1/2_ from 31 days to 10 days. As reported by Tavares et al. [42], storage temperature influences anthocyanin degradation due to their high thermosensitivity.

By comparing samples stored at 4 °C, we observed that the matrix did not have a protective effect against the degradation of TBC and AA, as the value of k was approximately 1.3 and 1.6 times higher for GG and AGG compared to CBJ, respectively. These differences in the value of k could be related to the available water content since CBJ had a high TSS (45 °Brix), exhibited a lower aw (0.911) than the GG and AGG samples (0.988 and 0.981, respectively). Thus, these conditions reduce the degradation of phenolic compounds in CBJ by decreasing the mobility of the reactants within the food [22,40]. In samples stored at 25 °C, a protective effect of gelatin was noticeable on the degradation of TBC and AA. A 52% and 38% lower k were observed in GM and AGM than in CBJ, respectively. Similar results were reported by Tutunchi et al. [22], who indicated that betanin was more stable in gummy candy than a beverage. In this regard, Rodriguez-Sánchez et al. [23] suggested that interactions with proteins may protect some pigments such as betaxanthins from hydrolysis and oxidation. Likewise, Muhamad et al. [48] indicated that sugar has a protective effect on anthocyanins, which could be assigned to its steric interference with reaction products of anthocyanin–phenolic polymerization [49]. The anthocyanins stability results showed a t_1/2_ of 16 and 12 days for GM and AGM, respectively. Tutunchi et al. [22] also observed a similar t_1/2_ in gummy candies enriched with red beet extract powder.

#### 2.2.2. Stability of Color

The CIELAB parameters (L*, a*, b*, and ΔE) of gelatin-based foods and CBJ stored for 35 days are shown in Table 4. 

**Figure 5:**
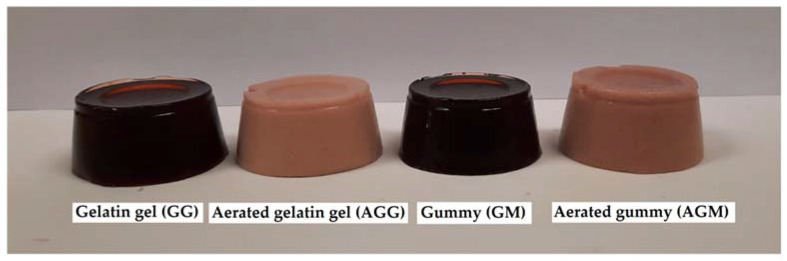
Photographs of the gelatin-based confectionery at day 0.

On day 0, CBJ had an intense reddish color mainly due to anthocyanins (L* ≈ 7.9, a* ≈ 24.56, and b* ≈ 4.91), and its addition explains the marked color of the samples, as can be seen in Figure 5. 

GG had low L* and higher a* compared to GM, indicating that GG presents a darker reddish color and may be related to a lower gelatin content in the formulation [24], highlighting the intense color of the CBJ. Meanwhile, the aerated samples (AGG and AGM) exhibited an opaque appearance with higher values of L* (*p* ≤ 0.05) compared to the non-aerated samples (GG and GM), which is mainly attributed to the air bubbles as reported by Zúñiga et al. [32] and Casas-Forero et al. [15]. In addition, the aeration produces an evident decrease in the darkness of the samples.

In terms of storage, a general decrease in L* values was observed, indicating a progressive darkening. On day 35, CBJ, GG, and AGG showed a decrease of 21%, 16%, and 14%, respectively, stored at 4 °C, while CBJ, GM, and AGM, which were kept at 25 °C, exhibited a reduction of 62%, 22%, and 28%, respectively. Thus, these results denote a protective effect of gelatin on color stability, as indicated by Otálora et al. [12] with gummy candies. Likewise, several authors have reported higher color stability in confectionery, such as gels and candies, made with gelatin as a gelling agent, compared to pectin [7,36,41]. Similarly, the coordinates of a* and b* decreased significantly with time storage (*p* ≤ 0.05), indicating that the samples lost their characteristic red coloration, which could be attributed to the degradation of anthocyanins in the chalcones [50].

The ΔE increased with time, reaching values greater than 5 units at 35 days. According to de Moura et al. [11], the human eye can distinguish the color change between samples with ΔE values greater than 3.5. The color difference was more noticeable in the CBJ stored at 25 °C (ΔE ≈18 units), whereas GG and GM exhibited lower values (ΔE ≈5 units). Comparable results were reported by Rivero et al. [9], de Moura et al. [11], and Yan et al. [51].

From a practical point of view, the present study can be useful in the food industry and molecular gastronomy, since the findings obtained are the use of the starting point for the cryoconcentrates used in gelling materials, which in turn allows multiple possibilities for food innovations.

## 3. Conclusions

The findings of this study suggest that it is possible to fortified gelatin-based confectionery with cryoconcentrated juice without detriment to their physicochemical characteristics and textural properties. CBJ improved nutritional value due to providing bioactive compounds with high antioxidant capacity. Specifically, on day 0, gelatin-based foods showed high TBC and AA. Regarding the stability study, a significant decrease in the TBC content and AA was observed in the CBJ and gelatin-based foods over storage (4 °C and 25 °C). Despite this, a protective effect of gelatin against loss of bioactive compounds and color changes was noticed. Furthermore, to preserve TBC, it can be estimated that GG and G could be stored for at least 21 days at 4 °C and 25 °C, respectively, while in aerated products (AGG and AGM), the storage time would be 15 days under storage at 4 °C and 25 °C, respectively. These findings support the idea that CBJ could be considered an innovative and functional ingredient. Furthermore, further studies must be carried out to evaluate the bioactivity of gelatin-based confectionery with cryoconcentrates to demonstrate their potential beneficial health effects.

## 4. Materials and Methods

### 4.1. Materials

Blueberries (*Vaccinium corymbosum* L.) were purchased from a local market in Chillán (36°36′24″ S 72°06′12″ W, XVI Región del Ñuble, Chile), and the fruits were stored at 4 °C until processing. Gelatin from bovine powder skin type B (G9382), sucrose, Folin–Ciocalteu reagent, gallic acid, cyanidin-3-glucoside, catequin, 1,1-diphenyl-2-picryl-hydrazyl (DPPH), 2,4,6-tris(2-pyridyl)-S-triazine (TPTZ), and 6-hydroxyl-2,5,7,8-tetramethyl-2-carboxylic acid (Trolox) were purchased from Sigma-Aldrich (St. Loius, MO, USA). Glucose syrup 42 DE was acquired locally from Furet Ltd. (Chillán, Chile). Standards and enzymes used to simulate in vitro digestion, such as human salivary α-amylase (1031), porcine pepsin (P6887), porcine pancreatin (H2625), and bovine bile (B3883), were purchased from Sigma-Aldrich (St. Loius, MO, USA). Calcium chloride (CaCl_2_), sodium carbonate (Na_2_CO_3_), ferric chloride hexahydrate (FeCl_3_·6H_2_O), sodium acetate (CH_3_COONa), sodium hydroxide (NaOH), hydrochloric acid (HCl), sodium nitrite (NaNO_2_), aluminum chloride (AlCl_3_), and potassium chloride (KCl) were obtained from Merck (Darmstadt, Germany). Ultrapure and distilled water were used for the preparation of all aqueous solutions. 

### 4.2. General Experimental Procedure

**Figure 6:**
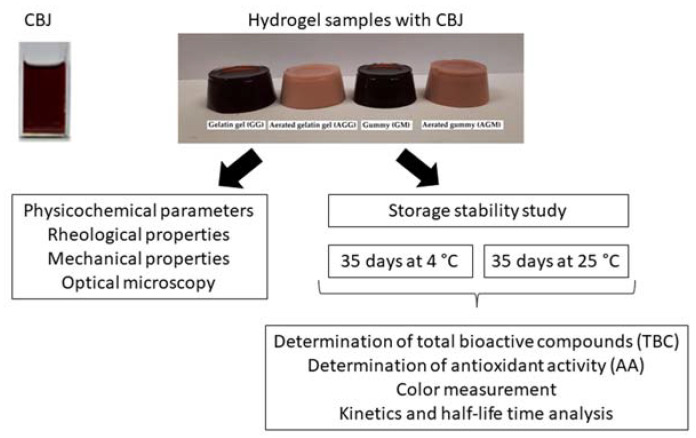
General experimental procedure.

Figure 6 shows the experimental procedure of the hydrogels fortified with CBJ, the characterization of hydrogels samples, storage stability, determination of total bioactive compounds (TBC) and antioxidant activity (AA), color measurement, and half-life time analysis.

### 4.3. CBJ Preparation

Blueberries were washed with tap water to remove dirt, and later, the juice was extracted in a tabletop juicer (JE2001, Nex, Barcelona, Spain). Immediately, the juice was filtered through a nylon cloth (0.8 mm mesh) to remove skin and seeds. Then, the juice was concentrated through three cryoconcentration cycles using the centrifugal method described previously by Casas-Forero et al. [16]. Hence, CBJ corresponds to the cryoconcentrated sample acquired in the final cycle. After the third cycle, the CBJ reached values of 45 °Brix and a 4.1 pH. Thus, the cryoconcentrated juice was stored until use as an ingredient in the elaboration of hydrogel samples. 

### 4.4. Preparation of Hydrogel Products

For the commercial hydrogels products based on confectionery foods, four types of samples were prepared, gelatin gel (GG), aerated gelatin gel (AGG), gummy (GM), and aerated gummy (AGM). For GG and AGG samples, the hydrogels with CBJ were performed according to Casas-Forero et al. [15], with slight modifications. Separately, the gelatin powder (3 g for GG and 8 g for AGG) was hydrated in 70 mL distilled water at room temperature for 10 min. Then, the solutions were mechanically stirred (300 rpm) for 10 min at 60 °C. After, the solutions were cooled and maintained for 5 min at 40 °C, and later, 30 g of CBJ was added to each solution, and the solution (with CBJ) was stirred (300 rpm) for 5 min at 40 °C. Later, the gel solution with CBJ was poured into plastic vessels (approximately 34 mm inner diameter and 15 mm height). For AGG samples, air bubbles were incorporated using an Oster^®^ mixer (Oster 2532, 250-watt, 6-speed, Rianxo, Spain) for 9 min, and then, the aerated gel solution with CBJ was deposited into plastic vessels. Finally, the GG and AGG samples were maintained overnight at 4 °C until the formation of a solid gel.

For GM and AGM samples, separately, 6 g of gelatin powder was hydrated in 25 mL distilled water for 10 min at room temperature. Then, the solution was heated at 60 °C and mechanically stirred at 300 rpm until complete dissolution (≈10 min). The gelatin solution was cooled and maintained until at 40 °C. In parallel, a syrup solution was prepared with 350 mL of distilled water, 350 mL of sucrose, and 300 mL of glucose syrup. Thus, once the solution was homogenized with constant agitation, it was heated for 5 min at 110 °C, and then, it was cooled to 50 °C. After, 30 g of CBJ and 45 g of syrup were mixed with the gelatin solution, and the mixture was stirred (300 rpm) for 5 min at 40 °C. Thus, for GM, the gel solution with CBJ and syrup was placed into plastic vessels. For AGM, the aeration process was performed for 4 min using an Oster^®^ mixer (Oster 2532, 250-watt, 6-speed, Rianxo, Spain). Finally, the aerated gel solution with CBJ and syrup was placed into plastic vessels. Thus, the GM and AGM were solidified overnight at room temperature.

### 4.5. Characterization of Hydrogels Samples

#### 4.5.1. Physicochemical Parameters

Total soluble solids (TSS) expressed as °Brix were analyzed using a digital PAL-1 refractometer (PAL-3, ≈1 mL, range: 0–93 °Brix, precision: ±0.1 °Brix, Atago Inc., Tokyo, Japan). The pH was measured using a digital pH meter (HI 2210, Hanna Instruments, Woonsocket, RI, USA). The moisture content was obtained gravimetrically by vacuum drying at 60 °C in a vacuum oven (3618–1CE, Lab Line Instruments Inc., Melrose Park, IL, USA) for 24 h (AOAC, 20.013, 2000). The water activity (*a_w_*) was determined using a dew-point hygrometer (AquaLab Model 4TE, Decagon Devices Inc., Pullman, WA, USA). The density was measured by the flotation method according to Zúñiga and Aguilera [31], and the results were expressed as kg/m^3^. Gas hold-up (*ε*) of AGG and AGM was obtained by comparing the density of the aerated sample (*ρ_AGG_* or *ρ_AGM_*) with the respective gas-free sample (*ρ_GG_* or *ρ_GM_*) density. The *ε* was calculated according to Equation (1).

(1) $$\varepsilon \left(\%\right)=\left(1-\frac{\rho_{AGG}\;or\;\rho_{AGM}}{\rho_{GG}\;or\;\rho_{GM}}\right)*100$$

where *ρ_AGG_*, *ρ_AGM_*, *ρ_GG,_* and *ρ_GM_* are the density of aerated gelatin gel (AGG), aerated gummy (AGM), gelatin gel (GG), and gummy (GM), respectively.

#### 4.5.2. Rheological Properties

The rheological properties were evaluated by a rotational-type rheometer (Physica MCR300, Anton Paar GmbH, Stuttgart, Germany) using a parallel plate geometry (50 mm diameter) with a 1 mm gap. The apparent viscosity of the samples was determined at 25 °C with a shear rate of 0.1 to 100 s^−1^ [27]. The frequency sweep measurement was performed from 0.1 to 100 Hz at a constant stress of 3.0 Pa within the linear viscoelastic region at 25 °C [52].

#### 4.5.3. Mechanical Properties

The uniaxial compression test was carried out using a texture analyzer (TAXT plus100, Stable Micro Systems Ltd., Surrey, UK) with a load cell of 5.0 kg. The samples were compressed with a 50 mm diameter cylindrical aluminum probe (P50) at a constant speed of 1 mm/s up to a compression strain of 70% [53]. The true stress (*σ_H_*) and true strain (*ε_T_*) were calculated from force-time curves by Equations (2) and (3), respectively.

(2) $$\sigma_{H}=F\;\left[\frac{h_{o}-h\;}{h_{o}\;A}\right]$$

(3) $$\varepsilon_{T}=-\ln\left[\frac{h_{o}}{h_{o}-h}\right]$$

where *F* is the compression force (N), *A* is the cross-sectional area of the sample (m^2^), and *h_o_* and *h* are the initial and final height after compression (m), respectively.

#### 4.5.4. Optical Microscopy

The samples were cut into thick slices of 3 mm with a razor blade and the slices were placed on a slide. Photomicrographs were acquired using an Olympus Trinocular Microscope (Olympus Co., Tokyo, Japan) coupled to a digital camera, Olympus LC 20 (Olympus Co., Munster, Germany) with an objective lens Nikon 10× [12].

### 4.6. Storage Stability Study (SSS)

Specifically, GG and AGG were maintained at 4 °C in the dark in a refrigerated incubator (FOC 215E, Velp Scientific Inc., Milano, Italy), and GM and AGM were stored at 25 °C in the dark in a thermostatic chamber (Memmert UF110, Memmert, Schwabach, Germany). For each sample and the control (CBJ), total bioactive compounds (TBC), antioxidant activity (AA), and color were analyzed at 0, 7, 14, 21, 28, and 35 days of storage.

### 4.7. Determination of Total Bioactive Compounds (TBC)

Total polyphenol content (TPC) was measured according to the Folin–Ciocalteu method described by Waterhouse [54], with minor modifications. Gallic acid (GA) was used as standard. An amount of 100 µL of the sample was mixed with 500 µL of 10-fold diluted Folin–Ciocalteu reagent. Then, the solution was vigorously mixed with 1500 µL of Na_2_CO_3_ (20% *w*/*v*). After 90 min in the dark at room temperature (incubation), the absorbance was recorded at 760 nm. TPC was calculated as milligrams of GA equivalents (GAE) per 100 g of sample (mg GAE/100 g). 

Total monomeric anthocyanin content (TAC) was quantified by the differential pH method according to Lee et al. [55], with some modifications. Cyanidin-3-O-glucoside (C3G) was used as standard. An amount of 200 µL of the sample was mixed with 800 µL of KCl (pH 1.0, 0.025 M) and 800 µL of CH_3_COONa (pH 4.5, 0.4 M) buffers. After 30 min in the dark at room temperature (incubation), the absorbance was measured at 510 and 700 nm. TAC was calculated as milligrams of C3G equivalents per 100 g of sample (mg C3G/100 g).

Total flavonoid content (TFC) was determined using the aluminum chloride colorimetric method described by Dewanto et al. [56], with modifications. Catequin (C) was used as standard. An amount of 250 µL of the sample was mixed with 1000 µL of distilled water and 75 µL of NaNO_2_ (5% *w*/*v*). After 6 min in the dark at room temperature (incubation), 75 µL of AlCl_3_ (10% *w*/*v*), 500 µL of NaOH (1 M), and 600 µL of distilled water were added to the solution, and later, the absorbance was measured at 510 nm. TFC was calculated as milligrams of C equivalents (CEQ) per 100 g of sample (mg CEQ/100 g).

### 4.8. Determination of Antioxidant Activity (AA)

The DPPH assay was assessed using the method reported by Brand-Williams et al. [57], with minor modifications. An amount of 150 µL of the sample was mixed with 2850 µL of DPPH methanolic solution. The mixture was kept in the dark at room temperature for 30 min (incubation), and the absorbance was measured at 515 nm.

The ferric reducing antioxidant power (FRAP) assay was performed according to Benzie and Strain [58], with some modifications. Briefly, FRAP reagent was prepared with 50 mL of CH_3_COONa buffer (pH 3.6, 300 mM), 5.0 mL of TPTZ (10 mM in HCl (40 mM)), and 5.0 mL of FeCl_3_·6H_2_O (20 mM) (10:1:1 ratio), and then the solution was incubated at 37 °C. An amount of 150 µL of the sample was mixed with 2850 µL of FRAP reagent. The solution was kept in the dark at 37 °C for 30 min (incubation), and the absorbance was measured at 593 nm.

For DPPH and FRAP assays, Trolox (T) was used as the standard curve, and the results were expressed as µmol Trolox equivalents (TE) per 100 g of sample (µM TE/100 g).

TBC (TPC and TAC) and AA (DPPH and FRAP) values were measured by spectrophotometric analysis (T70 UV/Vis spectrophotometer, Oasis Scientific Inc., Greenville, SC, USA).

### 4.9. Color Measurement

Color analysis was performed through CIELab coordinates (L*: darkness–lightness, a*: green-red axis, b*: blue-yellow axis) using a colorimeter (CM-5, Konica Minolta, Osaka, Japan), with illuminant D65 and an observer angle of 10°, where the samples were filled in a glass cuvette, and thus, the CIELab values were measured [59].

### 4.10. Kinetics and Half-Life Time Analysis

First-order kinetics was used to describe the degradation TBC and AA content [22]. The reaction rate constant (*k*) and half-life time (*t*_1/2_) were calculated according to Equations (4) and (5), respectively.

(4) $$\frac{C_{t}}{C_{o}}=e^{-k\;t}$$

(5) $$t_{1/2}=\frac{\ln2}{k}$$

where *C_o_* is the initial TBC and AA content, and *C_t_* is the TBC and AA content at time *t* (days).

### 4.11. Statistical Analysis

All experiments were replicated three times and measurements were carried out in triplicate, and the data were expressed as mean ± standard deviation (SD). A one-way analysis of variance (ANOVA) was performed for each variable to identify differences between samples, and least significant difference (LSD) tests were performed for the comparison of means at a significance level of 5% (*p* ≤ 0.05) using Statgraphics Centurion XVI software (v. 16.2.04, StatPoint Technologies Inc., Warrenton, VA, USA).

## Figures and Tables

**Table 1:** Physicochemical parameters of gelatin-based confectionery.

Physicochemical Parameter	GG	AGG	GM	AGM
TSS (°Brix)	16.8 ± 0.0 ^a^	21.0 ± 0.1 ^b^	61.0 ± 1.0 ^cd^	60.1 ± 0.2 ^c^
pH	5.0 ± 0.0 ^a^	5.2 ± 0.0 ^c^	5.1 ± 0.0 ^b^	5.1 ± 0.0 ^b^
Moisture (%)	83.1 ± 0.1 ^d^	78.9 ± 0.2 ^c^	38.7 ± 0.4 ^a^	38.3 ± 2.6 ^ab^
Water activity	0.988 ± 0.001 ^d^	0.981 ± 0.003 ^c^	0.889 ± 0.009 ^ab^	0.877 ± 0.008 ^a^
Density (kg/m^3^)	1083.2 ± 15.9 ^b^	408.6 ± 5.3 ^a^	1202.1 ± 33.1 ^c^	406.5 ± 14.2 ^a^
Gas hold-up (ε, %)	ND	62.3 ± 0.9 ^a^	ND	66.2 ± 1.5 ^b^

^a–d^: Different superscripts within the same row indicate significant differences at *p* ≤ 0.05. GG: gelatin gel; AGG: aerated gelatin gel; GM: gummy; AGM: aerated gummy; TSS: total soluble solid; ND: not determined.

**Table 2:** Total bioactive compounds (TBC) and antioxidant activity (AA) of the hydrogel samples.

Sample		TBC		AA	
	TPC	TAC	TFC	DPPH	FRAP
CBJ	773.3 ± 8.7 ^e^	22.3 ± 0.7 ^e^	566.8 ± 7.6 ^e^	4585.4 ± 8.5 ^e^	4442.0 ± 61.9 ^d^
GG	233.4 ± 1.6 ^a^	3.9 ± 0.1 ^cd^	136.1 ± 2.9 ^cd^	782.9 ± 3.0 ^b^	1067.8 ± 13.5 ^a^
AGG	251.0 ± 3.6 ^cd^	3.5 ± 0.2 ^a^	133.7 ± 3.7 ^c^	759.1 ± 1.1 ^a^	1057.3 ± 45.9 ^a^
GM	247.7 ± 0.9 ^c^	3.7 ± 0.2 ^abc^	119.6 ± 3.9 ^a^	911.3 ± 2.2 ^d^	1075.1 ± 12.6 ^ab^
AGM	238.5 ± 3.9 ^ab^	3.7 ± 0.1 ^ab^	123.9 ± 4.7 ^ab^	870.5 ± 4.8 ^c^	1083.3 ± 31.8 ^ab^

^a–e^: Different superscripts within the same column indicate significant differences at *p* ≤ 0.05. CBJ: cryoconcentrated blueberry juice; GG: gelatin gel; AGG: aerated gelatin gel; GM: gummy; AGM: aerated gummy.

**Table 3:** Kinetic parameters of total bioactive compounds and antioxidant activity in the CBJ and hydrogel samples stored under different conditions.

Assay	Kinetic Parameters	4 °C			25 °C		
		CBJ	GG	AGG	CBJ	GM	AGM
TPC	k (10^−3^ day^−1^)	13.0 ± 0.6 ^a^	18.6 ± 0.1 ^c^	24.9 ± 1.4 ^e^	36.8 ± 0.7 ^f^	16.4 ±0.6 ^b^	21.3 ± 1.3 ^d^
	t_1/2_ (day)	53.3 ± 2.7 ^f^	37.2 ± 0.3 ^d^	27.9 ± 1.6 ^b^	18.8 ± 0.4 ^a^	42.4 ± 1.5 ^e^	32.6 ± 1.9 ^c^
	R^2^	0.94	0.87	0.86	0.89	0.92	0.90
TAC	k (10^−3^ day^−1^)	22.3 ± 1.2 ^a^	40.9 ± 1.1 ^b^	47.9 ± 1.7 ^c^	67.7 ± 2.2 ^ed^	43.6 ± 2.4 ^b^	60.1 ± 7.2 ^d^
	t_1/2_ (day)	31.1 ± 1.8 ^e^	16.9 ± 0.4 ^d^	14.5 ± 0.5 ^c^	10.3 ± 0.3 ^a^	15.9 ± 0.9 ^cd^	11.5 ± 0.1 ^b^
	R^2^	0.97	0.99	0.97	0.97	0.98	0.99
TFC	k (10^−3^day^−1^)	21.8 ± 0.5 ^a^	28.4 ± 0.5 ^b^	38.8 ± 1.6 ^d^	66.7 ± 0.9 ^f^	33.1 ± 0.1 ^c^	41.9 ± 1.7 ^e^
	t_1/2_ (day)	31.8 ± 0.7 ^e^	24.4 ± 0.4 ^d^	17.9 ± 0.7 ^b^	10.4 ± 0.2 ^a^	20.9 ± 0.8 ^c^	16.5 ± 0.6 ^b^
	R^2^	0.96	0.98	0.95	0.97	0.90	0.99
DPPH	k (10^−3^ day^−1^)	16.3 ± 0.6 ^a^	21.2 ± 0.4 ^b^	25.6 ± 0.6 ^c^	40.2 ± 0.7 ^d^	21.3 ± 0.6 ^b^	26.2 ± 0.6 ^c^
	t_1/2_ (day)	42.5 ± 1.7 ^d^	32.8 ± 0.6 ^c^	27.1 ± 0.6 ^b^	17.2 ± 0.3 ^a^	32.6 ± 0.9 ^c^	26.5 ± 0.6 ^b^
	R^2^	0.98	0.90	0.96	0.98	0.96	0.97
FRAP	k (10^−3^ day^−1^)	24.7 ± 0.6 ^a^	31.7 ± 0.2 ^b^	37.9 ± 1.7 ^d^	54.2 ± 1.5 ^f^	34.1 ± 0.3 ^c^	41.7 ± 0.6 ^e^
	t_1/2_ (day)	28.0 ± 0.7 ^e^	21.9 ± 0.2 ^d^	18.2 ± 0.8 ^c^	12.8 ± 0.3 ^a^	20.3 ± 0.2 ^c^	16.6 ± 0.2 ^b^
	R^2^	0.96	0.98	0.94	0.97	0.97	0.99

^a–f^: Different superscripts within the same column indicate significant differences at *p* ≤ 0.05. CBJ: cryoconcentrated blueberry juice; GG: gelatin gel; AGG: aerated gelatin gel; GM: gummy; AGM: aerated gummy. TPC: Total polyphenol content, TAC: Total anthocyanin content, TFC: Total flavonoid content, k: kinetic degradation rate, t_1/2_: half-life time, R^2^: correlation index.

**Table 4:** Color parameters of CBJ and gelatin-based confectionery during storage.

Sample	Time (Days)	L*	a*	b*	ΔE
4 °C					
CBJ	0	8.0 ± 0.2 ^d^	24.6 ± 0.9 ^f^	4.9 ± 0.2 ^c^	-
	7	8.0 ± 0.0 ^d^	22.5 ± 0.0 ^e^	4.6 ± 0.1 ^c^	2.1 ± 1.0 ^a^
	14	7.1 ± 0.2 ^c^	21.6 ± 0.0 ^d^	4.3 ± 0.0 ^b^	3.1 ± 1.0 ^b^
	21	6.9 ± 0.2 ^bc^	21.0 ± 0.0 ^c^	4.3 ± 0.0 ^b^	3.8 ± 0.9 ^c^
	28	6.7 ± 0.2 ^b^	19.3 ± 0.1 ^b^	3.2 ± 0.1 ^a^	5.7 ± 0.9 ^d^
	35	6.3 ± 0.2 ^a^	17.4 ± 0.6 ^a^	3.2 ± 0.0 ^a^	7.6 ± 1.5 ^e^
GG	0	18.1 ± 0.6 ^d^	7.6 ± 0.4 ^f^	1.1 ± 0.0 ^d^	-
	7	17.2 ± 0.3 ^cd^	7.1 ± 0.2 ^e^	1.1 ± 0.1 ^d^	1.2 ± 0.4 ^a^
	14	16.6 ± 0.3 ^bc^	5.8 ± 0.2 ^d^	0.8 ± 0.1 ^c^	2.4 ± 0.2 ^b^
	21	16.2 ± 0.5 ^b^	4.5 ± 0.1 ^c^	0.7 ± 0.0 ^c^	3.8 ± 0.3 ^c^
	28	15.9 ± 0.6 ^ab^	4.1 ± 0.1 ^b^	0.6 ± 0.0 ^b^	4.3 ± 0.1 ^d^
	35	15.3 ± 0.2 ^a^	3.8 ± 0.1 ^a^	0.5 ± 0.0 ^a^	4.8 ± 0.4 ^e^
AGG	0	60.2 ± 0.5 ^e^	10.2 ± 0.2 ^f^	10.0 ± 0.1 ^d^	-
	7	58.7 ± 0.7 ^d^	9.6 ± 0.1 ^e^	9.9 ± 0.3 ^d^	1.7 ± 0.3 ^a^
	14	56.8 ± 0.3 ^c^	8.4 ± 0.2 ^d^	9.2 ± 0.0 ^c^	4.0 ± 0.0 ^b^
	21	54.8 ± 0.1 ^b^	7.9 ± 0.0 ^c^	8.6 ± 0.1 ^b^	6.0 ± 0.5 ^c^
	28	53.4 ± 0.1 ^a^	5.2 ± 0.0 ^b^	8.5 ± 0.0 ^b^	8.6 ± 0.2 ^d^
	35	51.9 ± 1.4 ^a^	4.6 ± 0.0 ^a^	7.7 ± 0.0 ^a^	10.3 ± 0.9 ^e^
25 °C					
CBJ	0	8.0 ± 0.2 ^f^	24.6 ± 0.9 ^f^	4.9 ± 0.2 ^e^	-
	7	6.8 ± 0.1 ^e^	18.4 ± 0.1 ^e^	3.9 ± 0.1 ^d^	6.4 ± 1.0 ^a^
	14	5.6 ± 0.0 ^d^	13.5 ± 0.5 ^d^	1.3 ± 0.0 ^c^	11.9 ± 1.3 ^b^
	21	4.6 ± 0.0 ^c^	10.7 ± 0.1 ^c^	1.0 ± 0.0 ^b^	14.8 ± 1.0 ^c^
	28	3.6 ± 0.0 ^b^	9.0 ± 0.0 ^b^	0.9 ± 0.0 ^a^	16.7 ± 1.0 ^d^
	35	3.0 ± 0.0 ^a^	7.6 ± 0.0 ^a^	0.9 ± 0.0 ^a^	18.1 ± 1.0 ^e^
GM	0	22.1 ± 0.6 ^e^	4.0 ± 0.1 ^f^	9.4 ± 0.4 ^d^	-
	7	21.0 ± 0.4 ^d^	2.8 ± 0.1 ^e^	9.7 ± 0.1 ^c^	1.7 ± 0.3 ^a^
	14	19.3 ± 0.1 ^c^	2.5 ± 0.1 ^d^	8.7 ± 0.3 ^b^	3.3 ± 0.5 ^b^
	21	18.6 ± 0.4 ^b^	2.4 ± 0.1 ^c^	8.8 ± 0.1 ^b^	3.9 ± 0.3 ^c^
	28	17.9 ± 0.2 ^a^	2.3 ± 0.0 ^b^	8.7 ± 0.3 ^b^	4.6 ± 0.4 ^d^
	35	17.3 ± 0.5 ^a^	1.7 ± 0.0 ^a^	8.1 ± 0.2 ^a^	5.5 ± 0.3 ^e^
AGM	0	56.4 ± 1.6 ^f^	11.3 ± 0.5 ^f^	9.4 ± 0.4 ^d^	-
	7	53.4 ± 0.8 ^e^	11.2 ± 0.1 ^e^	8.9 ± 0.1 ^c^	3.0 ± 1.0 ^a^
	14	47.8 ± 0.6 ^d^	10.9 ± 0.2 ^d^	8.8 ± 0.1 ^c^	8.6 ± 2.2 ^b^
	21	46.0 ± 1.0 ^c^	9.9 ± 0.3 ^c^	8.7 ± 0.3 ^bc^	10.5 ± 0.9 ^c^
	28	44.2 ± 0.5 ^b^	9.1 ± 0.0 ^b^	8.4 ± 0.1 ^b^	12.4 ± 1.6 ^d^
	35	40.7 ± 0.3 ^a^	8.8 ± 0.1 ^a^	7.9 ± 0.1 ^a^	15.9 ± 1.7 ^e^

^a–f^: Different superscripts within the same column indicate significant differences at *p* ≤ 0.05. CBJ: cryoconcentrated blueberry juice; GG: gelatin gel; AGG: aerated gelatin gel; GM: gummy; AGM: aerated gummy. TPC: Total polyphenol content, TAC: Total anthocyanin content, TFC: Total flavonoid content, k: kinetic degradation rate, t_1/2_: half-life time, R^2^: correlation index.

## Data Availability

The data is contained within the article.
